# Correlation between CT Value on Lung Subtraction CT and Radioactive Count on Perfusion Lung Single Photon Emission CT in Chronic Thromboembolic Pulmonary Hypertension

**DOI:** 10.3390/diagnostics12112895

**Published:** 2022-11-21

**Authors:** Toshiya Kariyasu, Haruhiko Machida, Tsuneo Yamashiro, Keita Fukushima, Masamichi Koyanagi, Kenichi Yokoyama, Makiko Nishikawa, Toru Satoh

**Affiliations:** 1 Department of Radiology, Faculty of Medicine, Kyorin University, 6-20-2 Shinkawa, Mitaka-shi 181-8611, Tokyo, Japan; 2 Department of Radiology, Tokyo Women’s Medical University Adachi Medical Center, 4-33-1 Kohoku, Adachi-ku 123-8558, Tokyo, Japan; 3 Diagnostic Radiology, Yokohama City University, 3-9, Fukuura, Kanazawa-ku, Yokohama-shi 236-0004, Kanagawa, Japan; 4 Department of Radiology, Kyorin University Hospital, 6-20-2 Shinkawa, Mitaka-shi 181-8611, Tokyo, Japan; 5 Department of Cardiology, Faculty of Medicine, Kyorin University, 6-20-2 Shinkawa, Mitaka-shi 181-8611, Tokyo, Japan

**Keywords:** chronic thromboembolic pulmonary hypertension, CT pulmonary angiography, lung subtraction CT, quantitative assessment

## Abstract

Background: Lung subtraction CT (LSCT), the subtraction of noncontrast CT from CT pulmonary angiography (CTPA) without spatial misregistration, is easily applicable by utilizing a software-based deformable image registration technique without additional hardware and permits the evaluation of lung perfusion as iodine accumulation, similar to that observed in perfusion lung single photon emission CT (PL-SPECT). The aim of this study was to use LSCT to newly assess the quantitative correlation between the CT value on LSCT and radioactive count on PL-SPECT as a reference and validate the quantification of lung perfusion by measuring the CT value in chronic thromboembolic pulmonary hypertension (CTEPH). Methods: We prospectively enrolled 47 consecutive patients with CTEPH undergoing both LSCT and PL-SPECT; we used noncontrast CT, CTPA, and LSCT to measure CT values and PL-SPECT to measure radioactive counts in areas representing three different perfusion classes—no perfusion defect, subsegmental perfusion defect, and segmental perfusion defect; we compared CT values on noncontrast CT, CTPA, and LSCT and radioactive counts on PL-SPECT among the three classes, then assessed the correlation between them. Results: Both the CT values and radioactive counts differed significantly among the three classes (*p* < 0.01 for all) and showed weak correlation (ρ = 0.38) by noncontrast CT, moderate correlation (ρ = 0.61) by CTPA, and strong correlation (ρ = 0.76) by LSCT. Conclusions: The CT value measurement on LSCT is a novel quantitative approach to assess lung perfusion in CTEPH and only correlates strongly with radioactive count measurement on PL-SPECT.

## 1. Introduction

Chronic thromboembolic pulmonary hypertension (CTEPH) results from the organization of thrombi and vascular remodeling in the pulmonary circulation that is associated with mean pulmonary artery pressure (mPAP) equal to or exceeding 25 mmHg [1,2,3,4]. It is generally screened using ventilation/perfusion (V/Q)-lung scintigraphy [5,6] or V/Q-lung single photon emission CT (SPECT) [7,8,9], which shows excellent sensitivity in detecting mismatched perfusion defects. High-resolution CT of the chest with lung window display, on the other hand, depicts inequalities in lung perfusion in a mosaic pattern of attenuation (MAP) in which areas of hypertransparency correspond with relatively decreased perfusion, a pattern commonly observed in CTEPH [10,11]. Confirmation of CTEPH is generally accomplished using CT pulmonary angiography (CTPA) with a multidetector CT scanner because this modality offers detailed information regarding typical morphological abnormalities of the pulmonary artery [5], whereas CTPA, and also catheter pulmonary angiography, is sometimes limited in patients with diffuse stenosis of the pulmonary artery. A single CTPA examination by dual-energy CT [12,13,14,15,16,17,18,19,20,21,22] or the subtraction of noncontrast from contrast-enhanced images without spatial misregistration in lung subtraction CT [23] permits evaluation of lung perfusion as iodine accumulation, similar to that observed in perfusion lung SPECT. Unlike dual-energy CT, which requires dedicated hardware to generate an iodine map, lung subtraction CT is easily applicable by utilizing a software-based deformable image registration technique without additional hardware. For qualitative assessment of CTEPH, Tamura et al. [23] described significantly higher diagnostic accuracy with lung subtraction CT than that with CTPA, with accuracy equivalent to that with perfusion lung SPECT. To our knowledge, however, no one has investigated quantification of lung perfusion in CTEPH with lung subtraction CT. The measurement of the CT value on lung subtraction CT may serve as a good objective surrogate marker of lung perfusion and is expected to play a critical role beyond its contribution to disease diagnosis in noninvasively strategizing optimal patient management based on detailed risk stratification in CTEPH. Therefore, the aim of this study was to newly assess a quantitative correlation of lung perfusion with lung subtraction CT using perfusion lung SPECT as a reference to validate the quantification of lung perfusion with lung subtraction CT by measuring the CT value.

## 2. Materials and Methods

### 2.1. Subjects

**Figure 1:**
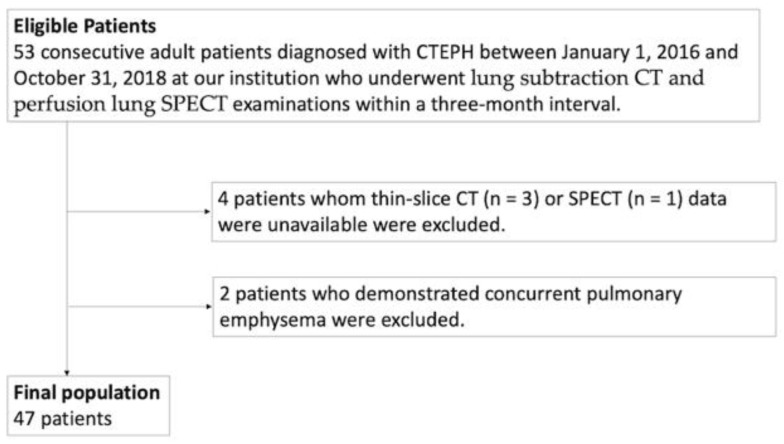
Flowchart of patient selection. CTEPH, chronic thromboembolic pulmonary hypertension; SPECT, single photon emission CT.

Between 1 January 2016 and 31 October 2018, at our institution, we identified 53 consecutive adult patients diagnosed with CTEPH who underwent lung subtraction CT and perfusion lung SPECT examinations within a three-month interval. These patients were diagnosed by an experienced cardiologist according to Nice guidelines [1], and had neither renal dysfunction nor a history of hypersensitivity to iodine contrast medium. Of the 53, we excluded 6 patients for whom thin-slice CT (*n* = 3) or SPECT (*n* = 1) data were unavailable or who demonstrated concurrent pulmonary emphysema (*n* = 2), finally including 47 patients (9 men, 38 women; mean age, 62 ± 14 years (range, 38 to 83 years)) in the study (Figure 1). Our initial exclusion criteria had also included patients who underwent other therapeutic interventions between the two examinations or manifested concurrent airway disease other than pulmonary emphysema or whose images showed significant motion artifacts from patient body movement and/or insufficient breath-hold; however, these criteria did not apply to any of our 53 patients. Table 1 summarizes patient demographic characteristics.

Our local ethics committee approved this prospective study (approval number: H27-120-05) and we obtained written informed consent from all subjects.

### 2.2. Image Acquisition Protocol

#### 2.2.1. Lung Subtraction CT

All patients were examined in the supine position using a 320-detector-row CT scanner (Aquilion ONE ViSION Edition; Canon Medical Systems, Tochigi, Japan). After the initial acquisition of noncontrast CT images of the chest that covered the entire lungs, patients were injected through a 20-gauge cannula in the right antecubital vein with iodine contrast medium containing 370 mg iodine/mL (Iopamilon 370; Bayer HealthCare, Osaka, Japan), which was administered as a fractional dose at 17.3 mg iodine/kg of body weight/second over 20 s. This was followed by the administration of a 40:60 mixture of contrast medium and saline over 10 s. CTPA scanning covering the entire lungs was started 25 s after the injection of contrast medium and performed in the caudocranial direction to avoid streak artifacts from highly concentrated contrast medium in the right subclavian vein or superior vena cava. Both noncontrast CT and CTPA were acquired with a *z*-axis automatic tube current modulation technique using the following parameters: tube voltage, 100 kV; rotation time, 0.275 s; collimation, 100 × 0.5 mm^2^; helical pitch, 0.81; and noise index, 25 HU, for the 5 mm reconstruction in filtered back-projection (FBP) with a standard kernel (FC03). We reconstructed a series of contiguous noncontrast CT and CTPA images of 1 mm slice thickness at 1 mm increments using a hybrid iterative reconstruction algorithm (Adaptive Iterative Dose Reconstruction (AIDR) 3D Standard, Canon Medical Systems), which is widely applicable in clinical settings.

To assess each patient’s exposure to radiation, we reviewed the volume CT dose index (CTDIvol) and dose-length product (DLP) recorded as a dose report and then calculated the estimated effective dose as the DLP multiplied by a k factor of 0.014 mSv·mGy^−1^·cm^−1^ for the chest.

#### 2.2.2. Perfusion Lung SPECT

All patients were examined in the supine position with a dual-head gamma-camera system (Symbia; Siemens Healthineers, Forchheim, Germany). Perfusion lung SPECT acquisition was started after a slow intravenous injection of 185-MBq Tc-labeled macroaggregated albumin (MAA) over two to three breathing cycles using the following parameters: energy window, 140.5 keV ± 15%; number of projections (per detector), 30; projection speed, 20 s per projection; detector configuration, 360 degrees; matrix size, 128 × 128; pixel size, 2.7 × 2.7 mm^2^; and slice thickness, 2.7 mm.

### 2.3. Fusion of Lung Subtraction CT and Perfusion Lung SPECT Images

**Figure 2:**
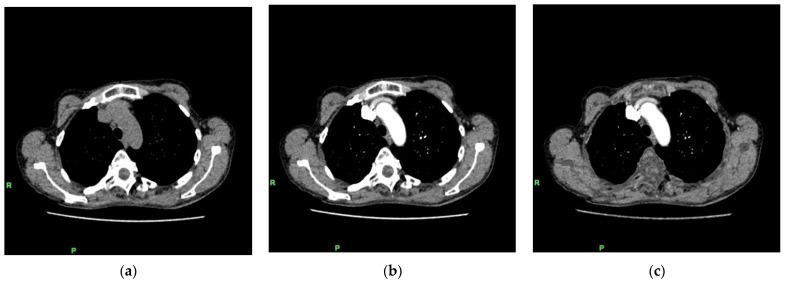
Lung subtraction CT generation process. Noncontrast chest CT axial image (**a**) and CTPA axial image (**b**) can be spatially matched based on deformable image registration by subtracting the noncontrast image from the contrast-enhanced image to generate a lung subtraction CT axial image (**c**). CTPA, CT pulmonary angiography.

**Figure 3:**
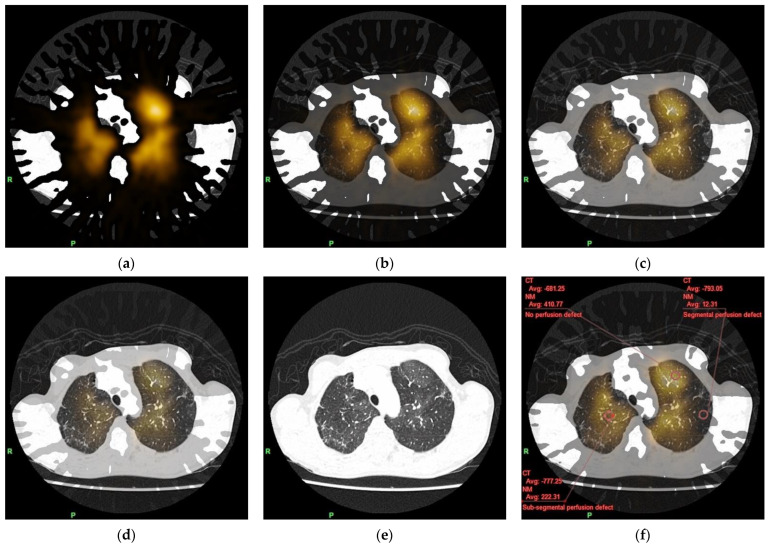
Fusion of lung subtraction CT and perfusion lung SPECT images at various blending ratios with and without ROIs placed in the three different classes of lung perfusion. Both a lung subtraction CT axial image at lung window display and a perfusion lung SPECT axial image can be fused by pixel-shift manual registration to eliminate spatial misregistration using the lung margin as the anatomical reference at various blending ratios, which can be arbitrarily determined from 0% ((**a**): perfusion lung SPECT only) to 100% ((**e**): lung subtraction CT only), including 25% (**b**), 50% (**c**), and 75% (**d**). Three circular ROIs of 1 cm^2^ can be manually placed in areas representing the three different classes of lung perfusion (Class 1, no perfusion defect; Class 2, subsegmental perfusion defect; and Class 3, segmental perfusion defect) on the axial fusion image at various blending ratios, such as 50% (**f**), for simultaneous (within each ROI) measurement of the CT value on lung subtraction CT and radioactive count on perfusion lung SPECT. ROI, region of interest; SPECT, single photon emission CT.

We transferred all the reconstructed axial noncontrast CT, CTPA, and perfusion lung SPECT images to a dedicated workstation (Ziostation2; Ziosoft, Inc., Tokyo, Japan), spatially matched the noncontrast CT and CTPA images of each patient based on deformable image registration, and subtracted the noncontrast images from the CTPA images to generate axial lung subtraction CT images (Figure 2). Both the axial lung subtraction CT and perfusion lung SPECT images were fused by pixel-shift manual registration using the lung margin for anatomical reference to eliminate spatial misregistration (Figure 3), while the fusion images were displayed at various blending ratios of lung subtraction CT/perfusion lung SPECT, which could be arbitrarily determined from 0% to 100% (Figure 3a–e). In this case, we used a blending ratio of 0% to represent perfusion lung SPECT only and 100% to represent lung subtraction CT only.

### 2.4. Image Evaluation

We excluded both the lung bases from evaluation because of their susceptibility to motion artifacts from cardiac pulsation and/or breathing, which can be similarly problematic also by dual-energy CT. By consensus, two board-certified radiologists subjectively identified three areas representing the different classes of lung perfusion in fusion images of each patient at a blending ratio of 0%. Class 1 represented no perfusion defect, Class 2 represented subsegmental perfusion defect, and Class 3 represented segmental perfusion defect. The readers manually placed a circular region of interest (ROI) of 1 cm^2^ in each area (a total of 141 ROIs in our 47 patients) for simultaneous measurement (within the same ROI) of the mean CT value on the noncontrast CT, CTPA, and lung subtraction CT images and the mean radioactive count on the perfusion lung SPECT images (Figure 3f). During this process, the readers observed MAPs on the fusion images at lung window display (window level, −500 HU; window width, 1500 HU) at a blending ratio of 100%, and as they changed blending ratios, they confirmed the increase of this regional transparency from Class 1 to Class 3, which well reflected the regional lung perfusion (Figure 3). At the same time, they carefully avoided streak artifacts from highly concentrated contrast medium in the right subclavian vein or the superior vena cava at mediastinum window display (window level, 50 HU; window width, 350 HU), as well as vessels and, if any, areas of focal change in attenuation (e.g., pulmonary infiltration) at lung window display at a blending ratio of 100%.

### 2.5. Statistical Analysis

Results are expressed as mean ± standard deviation (SD) for continuous variables. We analyzed the data using commercially available statistical software (SPSS for Windows, Version 23.0; IBM SPSS, Armonk, NY, USA), compared the mean CT value and the mean radioactive count among the three classes using a one-factor ANOVA test with Bonferroni correction, and correlated the mean CT value and the mean radioactive count using Spearman’s rank correlation coefficient. A *p* value below 0.05 was considered to indicate a significant difference.

## 3. Results

### 3.1. Radiation Exposure

In the single CT examination consisting of noncontrast CT and CTPA, the mean CTDI_vol_ was 4.3 ± 1.4 mGy, the mean DLP was 180.4 ± 55.0 mGy·cm, and the mean estimated effective dose was 2.5 ± 0.8 mSv.

### 3.2. Comparison of CT Value and Radioactive Count among the Three Different Classes of Lung Perfusion

**Figure 4:**
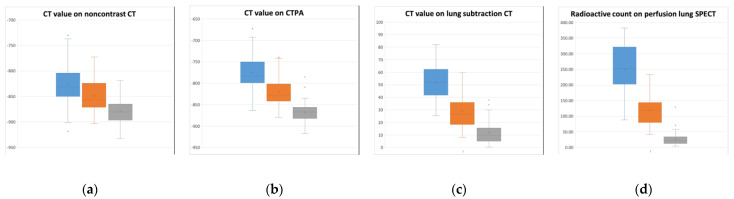
Box-and-whisker plots to compare CT value and radioactive count among different lung perfusion classes. CT values on noncontrast CT (**a**), CTPA (**b**), and lung subtraction CT (**c**) and radioactive count on perfusion lung SPECT (**d**) significantly decrease from Class 1 (blue: no perfusion defect) to Class 2 (orange: subsegmental perfusion defect) to Class 3 (gray: segmental perfusion defect) (*p* < 0.01 for all). The overlap of CT values among the three classes decreases from noncontrast CT to CTPA to lung subtraction CT (**a**–**c**). CTPA, CT pulmonary angiography; SPECT, single photon emission CT.

CT values on noncontrast CT (Figure 4a), CTPA (Figure 4b), and lung subtraction CT (Figure 4c) significantly decreased from Class 1 to Class 3 (*p* < 0.01 for all: *p* = 0.0051 for Class 1 vs. Class 2 on noncontrast CT; otherwise, *p* < 0.0001) (Table 2), and their overlap among the three classes decreased from noncontrast CT to CTPA to lung subtraction CT (Figure 3a–c). The radioactive count on perfusion lung SPECT also decreased significantly from Class 1 to Class 3 (*p* < 0.0001 for all) (Figure 3d).

### 3.3. Correlations between CT Value and Radioactive Count

**Figure 5:**
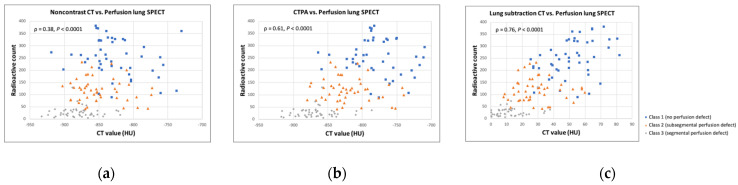
Correlations between CT value and radioactive count. Weak correlation is shown between CT value on noncontrast CT and radioactive count on perfusion lung SPECT (ρ = 0.38, *p* < 0.0001) (**a**); moderate correlation between CT value on CTPA and radioactive count (ρ = 0.61, *p* < 0.0001) (**b**); and strong correlation between CT value on lung subtraction CT and radioactive count (ρ = 0.76, *p* < 0.0001) (**c**). Blue plots represent Class 1 (no perfusion defect); orange, Class 2 (subsegmental perfusion defect); and gray, Class 3 (segmental perfusion defect). CTPA, CT pulmonary angiography; SPECT, single photon emission CT.

We observed a weak correlation between the CT value on noncontrast CT and radioactive count on perfusion lung SPECT (ρ = 0.38, *p* < 0.0001) (Figure 5a), a moderate correlation between the CT value on CTPA and radioactive count (ρ = 0.61, *p* < 0.0001) (Figure 5b), and a strong correlation between the CT value on lung subtraction CT (i.e., degree of contrast enhancement) and radioactive count (ρ = 0.76, *p* < 0.0001) (Figure 5c) (Table 3).

## 4. Discussion

In the present study, we first assessed the quantitative correlation of lung perfusion between lung subtraction CT and perfusion lung SPECT in CTEPH and observed a weak correlation between noncontrast CT and perfusion lung SPECT, a moderate correlation between CTPA and perfusion lung SPECT, and a strong correlation between lung subtraction CT and perfusion lung SPECT. Similar to radioactive counts on perfusion lung SPECT, CT values on noncontrast CT, CTPA, and lung subtraction CT decreased significantly from Class 1 to Class 3, while the overlap of CT values among the three classes decreased from noncontrast CT to CTPA to lung subtraction CT. Just as the map of iodine distribution generated by dual-energy CT reflects lung perfusion blood volume (PBV), the iodine map generated by lung subtraction CT allows accurate evaluation of abnormalities in lung perfusion in acute pulmonary embolism and CTEPH [13,16,20,23,24,25]. Using perfusion lung SPECT as a reference, Tamura et al. [23] described the high diagnostic accuracy of lung subtraction CT in the qualitative detection of defects in lung perfusion in patients with CTEPH. Noncontrast CT of the chest as well as CTPA with lung window display depict inequalities in lung perfusion as MAPs. In patients with CTEPH, these patterns reflect lung perfusion [10,11], but they may be overlooked because they can be very subtle [14]. Compared with both no contrast CT and CTPA, lung subtraction CT might more robustly reflect the lung perfusion in CTEPH, providing high-contrast iodine maps based on the subtraction of noncontrast images from contrast-enhanced images without spatial misregistration. Actually, during the image evaluation process, the readers observed MAPs on the fusion images at lung window display at a blending ratio of 100%, and as they changed blending ratios, they confirmed the increase of this regional transparency from Class 1 to Class 3. Similarly, a strong correlation has been described between dual-energy-CT-derived lung perfusion and MAPs [14]. Thus, lung subtraction CT could best differentiate the three classes of lung perfusion in CTEPH.

The functional and anatomical assessment of CTEPH lesions can be performed simultaneously by lung subtraction CT as well as dual-energy CT in a single comprehensive CTPA examination, allowing crucial early diagnosis. Surgical pulmonary endarterectomy (PEA) can provide a potential cure [26], and balloon pulmonary angioplasty (BPA) may prove beneficial in patients with technically inoperable disease or an unfavorable risk-to-benefit ratio for surgical PEA [27,28,29]. In BPA, target lesions should be selected based on lung perfusion and lesion morphology [30]. Lesions that allow visualization and assessment of peripheral vessels and branches appearing as “webs and bands” and demonstrating “abrupt narrowing” are better target candidates than lesions showing “chronic total occlusion” and “pouch defects” [31]. Hinrichs et al. [32] reported that pulmonary artery segments showing no signs of CTEPH that demonstrate preserved patency on C-arm CT during catheter pulmonary arteriography frequently correspond with the absence of perfusion defect (Class 1 in our study) on perfusion lung SPECT, whereas complete obstruction with segmental loss of pulmonary artery patency on C-arm CT corresponds well with segmental perfusion defect (Class 3 in our study) on perfusion lung SPECT. On the other hand, they rated subsegmental defects of pulmonary artery patency on C-arm CT almost equally as either no defect or subsegmental perfusion defect (Class 2 in our study) on perfusion lung SPECT [32]. The insufficient spatial resolution of most CT scanners can challenge the accurate detection of such subsegmental defects as partial obstruction, webs, and bands causing subsegmental filling defect [33,34].

Based on our results, lung subtraction CT may improve the detection of subsegmental defects in pulmonary artery patency and thereby improve the accurate diagnosis of CTEPH. It may also offer functional information regarding these lesions that suggests adequate treatment strategies, specifically, the restriction of BPA to patients with perfusion defects. Still, quantitative assessment of lung perfusion by lung subtraction CT may not replace qualitative assessment; however, future determination of a cut-off CT value on lung subtraction CT may aid the accurate diagnosis of CTEPH, adequate selection of target lesions before and during BPA, and optimal evaluation of lung reperfusion following BPA.

The mPAP obtained by right heart catheterization is one prognostic factor in patients with CTEPH [26,35,36]. Its correlation has been shown with quantitative assessment of lung PBV value by dual-energy CT as well as qualitative assessment of MAP [22,37,38]. According to Derlin et al. [39], quantitative assessment of the extent of perfusion defect on perfusion lung SPECT/CT (sensitivity, 88%; specificity, 64%) and lung PBV value on dual-energy CT (sensitivity, 78%; specificity, 87%) enabled diagnosis of mPAP above 50 mmHg in CTEPH. Thus, quantitative assessment of whole lung perfusion by lung subtraction CT may also be useful as a noninvasive means to estimate the clinical severity of CTEPH and identify patients with high-risk disease, avoiding hospitalization and reducing medical costs.

Compared with dual-energy CT, lung subtraction CT utilizes motion correction software without the need for additional hardware, which facilitates its clinical implementation and reduces its cost. In addition, a lung subtraction CT approach that utilizes the entire difference in tissue attenuation between noncontrast and contrast-enhanced images has been reported to offer a higher contrast-to-noise ratio than that obtained by dual-energy CT using the assessment of spectral decomposition between the tissue and iodine [40,41]. Observer preference was also reported for lung subtraction CT with its higher signal-to-noise ratio at a lower total radiation dose than that with dual-energy CT [24]. Although lung subtraction CT requires the acquisition of noncontrast CT images and dual-energy CT does not, our patients were exposed to a lower or comparable total radiation dose by lung subtraction CT by our use of a low-dose protocol (tube voltage, 100 kV; noise index, 25 HU) and a hybrid iterative reconstruction algorithm, which is widely applicable in clinical settings. In contrast, the reported results by dual-energy CT were: CTDI_vol_, 5.4–10.8 mGy; DLP, 161–400 mGy·cm; and estimated effective dose, 2.3–6.8 mSv [13,14,16,17,18,19,20,21,22,23].

Our study has several limitations. Firstly, CTEPH is fairly uncommon, so our study included only a small population at a single institution. Secondly, patient positioning may have influenced the distribution of the radioactive tracer throughout the lungs and our results. Patients were placed in the supine position prior to injection with Tc-labeled MAA, and perfusion lung SPECT was examined without respiratory gating, so we evaluated neither lung base because of their susceptibility to respiration-related motion artifacts, which can be similarly problematic also by dual-energy CT. Although the lung bases are the predominant site of CTEPH, our purpose was to assess a quantitative correlation of lung perfusion between lung subtraction CT and perfusion lung SPECT. Different from other SPECT examinations, normalization of lung perfusion quantification was difficult using other organs for reference. Finally, discrepant quantification of lung perfusion between lung subtraction CT and perfusion lung SPECT may have resulted from compensatory perfusion to the lungs via systemic collaterals, such as the bronchial arteries, which often occurs in patients with CTEPH. Whereas bolus tracking or the test injection technique was not applied, CTPA scanning was constantly timed to begin 25 s after contrast-medium injection, which is optimal to achieve strong contrast enhancement solely in the pulmonary circulation. On the other hand, 25 s may have been too early to obtain sufficient contrast enhancement via the systemic collaterals. However, further studies, including a large study population at multiple institutions, are needed to not only confirm our results also by bolus tracking or the test injection technique in CTPA examinations and using perfusion lung SPECT with respiratory gating as a reference, but also evaluate the clinical usefulness of this quantitative approach for appropriate management of patients with CTEPH.

## 5. Conclusions

We first applied the measurement of the CT value on lung subtraction CT without additional hardware as a novel quantitative approach to assess lung perfusion in patients with CTEPH. This approach correlates strongly with the measurement of radioactive count on perfusion lung SPECT as a reference standard, and allows sensitive differentiation among the three classes of lung perfusion–no perfusion defect, subsegmental perfusion defect, and segmental perfusion defect. Lung subtraction CT may be used to noninvasively diagnose disease and plays a critical role in optimal decision making, such as adequate determination of indications for BPA and optimal evaluation of lung reperfusion following BPA, in the management of patients with CTEPH.

## Figures and Tables

**Table 1:** Patient demographic characteristics.

Number of Patients	47
Sex	Men, 9 (19.1%); women, 38 (80.9%)
Age (range)	62 ± 14 years (38–83 years)
BMI (range)	23.6 ± 3.9 kg/m^2^ (15.8–33.2 kg/m^2^)
WHO-FC	I (*n* = 2), II (*n* = 41), III (*n* = 4), IV (*n* = 0)
Mean PA pressure (range)	25.4 ± 16.8 mmHg (10–113 mmHg)
Prior therapy	Medication only (*n* = 6) Medication with PEA (*n* = 1) Medication with BPA (*n* = 40)

BMI, body mass index; BPA, balloon pulmonary angioplasty; PEA, pulmonary endarterectomy; WHO-FC, World Health Organization functional class.

**Table 2:** CT value and radioactive count among different classes of lung perfusion.

	Noncontrast CT	CTPA	Lung Subtraction CT	Perfusion Lung SPECT
Class 1	−826.6 ± 40.6	−774.8 ± 40.1	51.9 ± 14.5	250.1 ± 76.3
Class 2	−848.9 ± 33.1	−819.8 ± 34.6	29.1 ± 13.1	119.1 ± 47.9
Class 3	−879.3 ± 25.8	−867.6 ± 27.3	11.7 ± 9.3	26.5 ± 20.9

Data are mean ± standard deviation (SD). All CT values (HU) and radioactive counts differ significantly among the different perfusion classes (*p* < 0.01 for all). CTPA, CT pulmonary angiography; SPECT, single photon emission CT.

**Table 3:** Correlations between CT value and radioactive count.

	ρ Value	*p* Value
Noncontrast CT vs. Perfusion lung SPECT	0.38	<0.0001
CTPA vs. Perfusion lung SPECT	0.61	<0.0001
Lung subtraction CT vs. Perfusion lung SPECT	0.76	<0.0001

CTPA, CT pulmonary angiography; SPECT, single photon emission CT.

## Data Availability

Not applicable.
